# Skin prick test reactivity to lupin in comparison to peanut, pea, and soybean in atopic and non-atopic German subjects: A preliminary cross-sectional study

**DOI:** 10.1002/iid3.24

**Published:** 2014-06-03

**Authors:** Melanie Bähr, Anita Fechner, Martin Kaatz, Gerhard Jahreis

**Affiliations:** 1 Department of Nutritional Physiology, Institute of Nutrition, Friedrich Schiller University JenaDornburger Str. 24, 07743, Jena, Germany; 2 SRH Wald-Klinikum Gera GmbH, Zentrum für klinische StudienStrasse des Friedens 122, 07548, Gera, Germany

**Keywords:** Atopy, cross-reactivity, legumes, lupin, pea, peanut, sensitization, soybean

## Abstract

The increasing use of lupin in food processing poses a problem of potential (cross-)allergic reactions. To evaluate the prevalence of sensitization to lupin in comparison to that of other legumes skin prick tests were performed with lupin, pea, peanut, and soybean in atopic (*n* = 81) and non-atopic (*n* = 102) German adults. Of these 183 subjects, 20 subjects had to be excluded due to invalid skin prick tests (reaction to histamine <3 mm or to sodium chloride >2 mm). Thus, skin prick tests of 163 subjects were included in final analyses. Of 163 subjects, 18 had a positive reaction to at least one legume tested. Overall skin prick test reactivity was different among non-atopic and atopic subjects (*P* = 0.005). Altogether, six subjects (4%) were sensitized to lupin, 12 (7%) to pea, 5 (3%) to peanut, and 8 (5%) to soybean. Two (2%) of the 92 non-atopic subjects and 4 (6%) of the 71 atopic subjects had a positive skin prick test to lupin. Of the 6 subjects sensitized to lupin, 3 (50%) were also sensitized to pea, 3 (50%) to peanut, and 5 (83%) to soybean. In conclusion, the prevalence rates of lupin sensitization were comparable to or even lower than those of pea, peanut, and soybean. To date, lupin allergy is suspected to be relatively uncommon in the overall German population since lupin sensitization occurred in only 2% of non-atopic subjects. However, there is a clear risk of a lupin allergy in predisposed subjects, since the frequency of lupin sensitization was 6% in atopic subjects. In particular, subjects with existing sensitization or allergy to other legumes are at higher risk for a sensitization or allergy to lupin due to cross-reactivity.

## Introduction

The use of plant proteins, such as lupin protein in human nutrition is associated with various physiological, ecological, and technological benefits. Lupin flour is incorporated in different commercial food products serving as an additive or substitute to wheat flour in, not only, but also gluten-free bakery products and foods for celiac disease patients [1]. The increasing use of lupin in food processing presents a problem of potential unspecific allergic reactions since lupin then is present as a hidden food allergen. Thus, in December 2006, lupin and products thereof were included in the Annex IIIa of Directive 2000/13/EC, which contains ingredients that must appear on the labeling of foodstuffs [2].

Since lupin belongs to the *Fabaceae* family, the frequency of cross-allergies between lupin and other legumes might be high. Hefle et al. [3] first observed a high rate of cross-allergy with 5 (71%) positive skin prick tests (SPTs) to lupin in 7 adult patients allergic to peanut. Moneret-Vautrin et al. [4] detected 44% (11 of 24) positive SPTs to lupin in peanut-allergic children. In an oral challenge study, the cross-allergic reaction to lupin concerned 68% of the 23 peanut-allergic patients [5]. In a study from Shaw et al. [6], 16 out of 47 (34%) peanut-allergic children were sensitized to lupin. According to Smith et al. [7], approximately 19–25% of the peanut-allergic individuals are sensitized to lupin, however, only 6–8% react with clinically relevant symptoms to lupin. The Federal Institute for Risk Assessment in Germany [8] states that the risk of a crossed peanut-lupin allergy is about 30–60%. Around 1% of the UK population suffers from peanut allergy and up to half of it could be presensitized or allergic to lupin [9]. In contrast to lupin-peanut cross-reactivity, the data on lupin-soybean and lupin-pea cross-reactions are scarce. Furthermore, the prevalence rates of a lupin sensitization in the overall German population are not known. Previous studies referred to predisposed subjects who, thus, are more likely of having sensitization or allergy to legumes than the average population. The frequency of a sensitization to lupin found in these selected patient groups does not reflect the prevalence referring to the overall population. Thus, the aim of the present study was to evaluate the prevalence of a sensitization to lupin (positive SPT) in comparison to that of other legumes (pea, peanut, and soybean) in atopic and non-atopic German subjects.

## Materials and Methods

### Study population

A total of 208 volunteers were assessed for eligibility. Exclusion criteria were treatment with antihistamines, tricyclic antidepressants, systemic corticosteroids, or topical corticosteroids used at the tested skin area. Furthermore, subjects with infections of the skin at the test area, subjects with acute inflammatory status, and those with allergic symptoms as well as breast-feeding mothers and pregnant females were excluded. Finally, 183 participants (128 females, 55 males) were invited to an in-person meeting with a physician. Here, participants were offered essential study-relevant information in both oral and written form. Subjects gave written informed consent and then underwent administration of a comprehensive questionnaire and a SPT conducted by a physician.

Study participants were assigned to one of two study groups: (1) non-atopic subjects and (2) atopic subjects. For the latter the inclusion criteria were one or more of the following: documented allergy, neurodermatitis, allergic asthma, allergic rhinitis, and/or a positive atopy score (>10 points) based on the criteria of Erlangen (see below). The study was registered at ClinicalTrials.gov as NCT01728168 (National Institutes of Health) and approved by the ethics committee of the State Chamber of Physicians of Thuringia (no.: 35482/2012/131).

### Questionnaire

The questionnaire consisted of a case report form, including information about personal and anthropometric data, concomitant medication, and questions according to the criteria of Erlangen. The Erlanger atopy score focuses on atopic dermal diathesis such as atopic dermatitis and includes questions addressing the atopic history of the family, personal anamnesis, atopic minimal forms and atopic eczema, stigmata of atopic constitutions as well as the dermal neurovegetative system. The score does not consider respiratory atopies such as allergic rhinitis or allergic asthma in detail.

### Skin prick test (SPT)

SPTs were performed with *Pisum sativum* and *Arachis hypogaea* (pea and peanut, 5000 protein nitrogen units/mL, Allergopharma GmbH & Co. KG, Reinbeck, Germany), *Glycine max* (soybean, 1/20 w/v, ALK Abelló Arzneimittel GmbH, Hamburg, Germany) and NaProLup PO54, a commercial protein-fiber compound from *Lupinus albus* (lupin, NaProFood, GmbH & Co. KG, Bruckberg, Germany). Histamine dihydrochloride and sodium chloride served as positive control and negative control, respectively (ALK Abelló Arzneimittel GmbH). The SPT for lupin was conducted by mixing NaProLup PO54 with the negative control directly on the skin and pricking through the material. All test solutions were stored at 4°C. SPTs were performed according to the local protocol of the SRH Wald-Klinikum Gera GmbH. Single drops were put on the flexor side of the forearm. To avoid direct contamination of one test by another not more than four droplets on one forearm were applied while keeping a distance of at least 3.5 cm between droplets. A lancet was used to prick through the droplets. SPT reactions were read after 20 min. SPTs with a wheal diameter of <3 mm elicited by histamine dihydrochloride or with a wheal diameter of >2 mm elicited by sodium chloride were considered invalid and were thus excluded. Positive SPT reactivity was defined as the presence of a mean wheal diameter of >3 mm. Wheals with a diameter of 2–3 mm were considered potentially positive. In the case of an erythema, the SPT was considered positive with a wheal diameter of >2 mm.

### Statistics

Statistical analyses were conducted using SPSS 19.0 (SPSS Inc., Chicago, Illinois, USA). In all statistical analyses, *P* was considered significant when ≤0.05. Due to the small sample sizes, Fisher's exact test was applied to detect differences in prevalence rates between allergens or between atopic and non-atopic subjects. Estimations of confidence intervals were conducted using Monte Carlo simulation (95% CI). The posteriori *z*-test for comparing column proportions was applied for differences in overall SPT reactivity between atopic and non-atopic subjects as well as for differences in SPT reactivity between allergens. For analysis of the level of coincidence in the SPT reactions among different allergens, kappa coefficients were calculated including positive and negative SPT reactions.

## Results

### Baseline characteristics

Of the 183 subjects, 20 subjects had invalid SPTs due to a missing reaction to histamine dihydrochloride (*n* = 9) or a positive reaction to sodium chloride (*n* = 11). Hence, the SPTs of 163 subjects were included in the final analysis. One hundred and two non-atopic subjects were screened, of whom 10 had to be excluded due to an invalid SPT. The mean Erlanger atopy score of the remaining 92 non-atopic subjects was 0.9 ± 1.7 (Table1). Eighty-one atopic subjects were screened, of whom 10 were excluded due to an invalid SPT. The mean Erlanger atopy score of the remaining 71 atopic subjects was 4.4 ± 4.7. The Erlanger atopy score was relatively low in atopic subjects, since it only included subjects with atopic dermal diathesis but not those with respiratory atopy.

**Table 1:** Baseline data of non-atopic and atopic subjects

	Non-atopic subjects	Atopic subjects
*n*	*92*	*711*
Females	*68* (74%)	*47* (66%)
Males	*24* (26%)	*24* (34%)
Age	44.1 ± 16.5	41.7 ± 15.3
Height	169.0 ± 8.8	170.7 ± 8.6
Body weight (kg)	69.7 ± 16.1	71.8 ± 12.6
BMI (kg/m^2^)	24.3 ± 5.0	24.6 ± 3.7
Erlanger atopy score	0.9 ± 1.7	4.4 ± 4.7
Documented allergy	*0* (0%)	*30* (42%)
Allergic asthma or rhinitis	*0* (0%)	*46* (65%)
Positive atopy score*	*0* (0%)	*11* (16%)

Figures represent mean ± SD or *numbers* (%) of subjects.

BMI, body mass index.

* Based on the criteria of Erlangen an atopy score >10 points is defined as a positive atopy score.

### Overall SPT reactivity

Of the total cohort with valid SPTs (*n* = 163), 18 subjects (11%) had positive SPT reactions to at least one of the legumes tested. Forty-nine subjects (30%) had potentially positive SPT reactions, whereas 96 subjects (59%) had negative SPTs.

**Figure 1:**
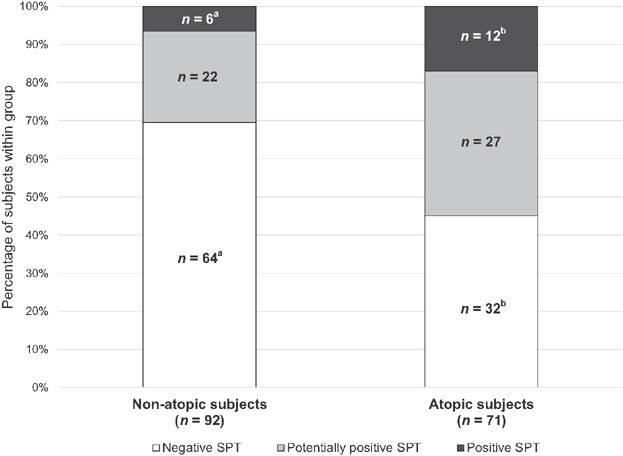
Overall SPT reactivity in non-atopic and atopic subjects. SPT, skin prick test; ^a,b^Different superscript letters indicate significant differences between atopic and non-atopic subjects within one category (*z*-test, significant for *P* ≤ 0.05).

The overall SPT reactivity was different among non-atopic and atopic subjects with *P* = 0.005 (95% CI: 0.003–0.006). The *z*-test for comparing column proportions revealed a significantly higher frequency of negative SPTs and a significantly lower prevalence of positive SPTs in non-atopic compared to atopic subjects (*P* ≤ 0.05, Fig. 1).

### Allergen-specific SPT reactivity

Of the subjects with valid SPTs (*n* = 163), 12 (7%) had positive SPTs to pea, 5 (3%) to peanut, 8 (5%) to soybean, and 6 (4%) to lupin (Table2). No significant difference was found for SPT reactivity between pea, peanut, soybean, and lupin. However, the *z*-test for comparing column proportions revealed a significantly higher frequency of negative SPTs to peanut compared to pea (*P* ≤ 0.05).

**Table 2:** Results of SPTs to each allergen in the total cohort (*n* = 163)

		Pea	Peanut	Soybean	Lupin		*P*-value* (95% CI)
P^−^	*n*	*121*^a^	*137*^b^	*130*^ab^	*131*^ab^	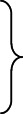	0.411 (0.401–0.420)
	%	74.2	84.0	79.8	80.4		
P^?^	*n*	*30*	*21*	*25*	*26*		
	%	18.4	12.9	15.3	16.0		
P^+^	*n*	*12*	*5*	*8*	*6*		
	%	7.4	3.1	4.9	3.7		

Figures represent *numbers* or precentage of subjects.

SPT, skin prick test; P^−^, negative SPT; P^?^, potentially positive SPT; P^+^, positive SPT.

* Monte Carlo significance (2-sited) with the Fisher's exact test (significant for *P* ≤ 0.05) for differences in prevalence rates of SPT reactivity including negative, potentially positive, and positive SPTs between pea, peanut, soybean, and lupin.

^a,b^Different superscript letters indicate significant differences between allergens within a row (*z*-test, significant for *P* ≤ 0.05).

The SPT reactivity was significantly different between non-atopic and atopic subjects for pea and tended to be different for soybean, whereas for peanut and lupin no significant differences were observed (Table3).

**Table 3:** Results of SPTs to each allergen in non-atopic (*n* = 92) and atopic subjects (*n* = 71)

		Pea		Peanut		Soybean		Lupin	
					
		Non-atopic	Atopic	Non-atopic	Atopic	Non-atopic	Atopic	Non-atopic	Atopic
P^−^	*n*	*78*	*43*	*81*	*56*	*79*	*51*	*76*	*55*
	%	84.8	60.6	88.0	78.9	85.9	71.8	82.6	77.5
P^?^	*n*	*12*	*18*	*9*	*12*	*10*	*15*	*14*	*12*
	%	13.0	25.4	9.8	16.9	10.9	21.1	15.2	16.9
P^+^	*n*	*2*	*10*	*2*	*3*	*3*	*5*	*2*	*4*
	%	2.2	14.1	2.2	4.2	3.3	7.0	2.2	5.6
		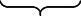		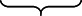		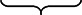		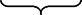	
*P*-value*		0.001		0.313		0.079		0.536	
(95% CI)		0.0003–0.001		0.304–0.322		0.074–0.084		0.526–0.545	

Figures represent *numbers* or precentage of subjects.

SPT, skin prick test; P^−^, negative SPT; P^?^, potentially positive SPT; P^+^, positive SPT.

* Monte Carlo significance (2-sited) with the Fisher's exact test (significant for *P* ≤ 0.05) for differences in prevalence rates of SPT reactivity including negative, potentially positive, and positive SPTs between non-atopic and atopic subjects.

### Cross-reactivity among legumes

Of the 6 subjects sensitized to lupin, 3 (50%) had positive SPTs to pea, 3 (50%) had positive SPTs to peanut, and 5 (83%) had positive SPTs to soybean (Table4).

**Table 4:** Results of SPTs to pea, peanut, and soybean dependent on the SPT reactivity to lupin

*n* = 163		Lupin P^−^ (*n* = 131)			Lupin P^?^ (*n* = 26)			Lupin P^+^ (*n* = 6)		
				
	Total *n*	*n*	(% of column)	(% of row)	*n*	(% of column)	(% of row)	*n*	(% of column)	(% of row)
Pea P^−^	*121*	*107*	81.7	88.4	*13*	50.0	10.7	*1*	16.7	0.8
Pea P^?^	*30*	*19*	14.5	63.3	*9*	34.6	30.0	*2*	33.3	6.7
Pea P^+^	*12*	*5*	3.8	41.7	*4*	15.4	33.3	*3*	50.0	25.0
Peanut P^−^	*137*	*117*	89.3	85.4	*19*	73.1	13.9	*1*	16.7	0.7
Peanut P^?^	*21*	*14*	10.7	66.7	*5*	19.2	23.8	*2*	33.3	9.5
Peanut P^+^	*5*	*0*	0.0	0.0	*2*	7.7	40.0	*3*	50.0	60.0
Soybean P^−^	*130*	*113*	86.3	86.9	*16*	61.5	12.3	*1*	16.7	0.8
Soybean P^?^	*25*	*15*	11.5	60.0	*10*	38.5	40.0	*0*	0.0	0.0
Soybean P^+^	*8*	*3*	2.3	37.5	*0*	0.0	0.0	*5*	83.3	62.5

Figures represent *numbers* or precentage of subjects.

SPT, skin prick test; P^−^, negative SPT; P^?^, potentially positive SPT; P^+^, positive SPT.

Of the 12 subjects sensitized to pea, 3 (25%) had positive and 4 (33%) had potentially positive SPTs to lupin. Of the 5 subjects sensitized to peanut, 3 (60%) had positive, and 2 (40%) had potentially positive SPTs to lupin. Of the 8 subjects sensitized to soybean, 5 (63%) had positive and none (0%) had potentially positive SPTs to lupin.

### Gender- and age-specific prevalence

None of the 48 male subjects had a positive SPT to peanut or lupin, whereas within the 115 females, 5 (4%) had positive SPTs to peanut and 6 (5%) had positive SPTs to lupin. Positive SPTs to pea were observed in 3 of 48 (6%) males and 9 of 115 (8%) females. Positive SPTs to soybean were observed in 3 of 48 (6%) males and 5 (4%) of 115 females. No significant difference between genders was found in the prevalence rates for SPT reactivity neither for pea, peanut, soybean, nor for lupin (data not shown). Considering age, no significant difference was found in prevalence rates for SPT reactivity between younger (≤43 years, *n* = 86) and older subjects (>43 years, *n* = 77; data not shown).

## Discussion

### Prevalence of lupin sensitization

This study examined for the first time the SPT reactivity to lupin by including non-atopic German subjects. The results enable a first rough estimate of the current prevalence rates of sensitization to lupin in the overall German population. The study reveals a lupin sensitization rate of 2% in non-atopic and 6% in atopic subjects. These rates were comparable to or even lower than those of pea (2% and 14%), peanut (2% and 4%), and soybean (3% and 7%) observed in non-atopic and atopic subjects, respectively.

Between 2005 and 2006, Reis et al. [10] examined patients of the Mediterranean area consulting allergologists and found a lupin sensitization rate of 4% (48 of 1160). Shaw et al. [6] found 2 of 46 (4%) atopic subjects of Great Britain to be sensitized to lupin. In a Norwegian study [11], 15 of 35 (43%) food-allergic children had a positive SPT to lupin. An investigation in French and Belgian subjects revealed a sensitization to lupin in 6 of 990 (0.6%) non-atopic adults and in 53 of 1422 (4%) atopic adults [12]. In the study of de Jong et al. [13], 6% (22 of 372) of the examined Dutch patients with a suspected food allergy had positive SPTs to lupin flour, whereas the sensitization rate to peanut and soybean was higher with 36% (135 of 372) and 16% (58 of 372), respectively. The heterogeneity of the above-mentioned study cohorts regarding the level of predisposition (e.g., non-atopic subjects, individuals consulting allergologists, atopic subjects, and food-allergic subjects) constrains the comparability of the prevalence rates of a lupin sensitization.

### Clinical relevance of lupin sensitization

The prevalence of a sensitization to a specific food allergen does not reflect the frequency of a clinically relevant allergy. Whereas in a study of Moneret-Vautrin et al. [4], 7 of 8 (90%) lupin-sensitized subjects who underwent oral challenge to lupin flour showed positive responses, Peeters et al. [14] found 8 of 23 (35%) peanut-sensitized adults to suffer from a clinically relevant lupin sensitization. Shaw et al. [6] found similar results: 2 of 9 (22%) peanut-allergic children sensitized to lupin reacted to oral challenge with lupin. In the study of Lindvik et al. [11], of 10 food-allergic children with positive SPT, only one (10%) reacted with urticaria, angioedema, and cough when challenged orally with lupin flour. Similarly, in the study of de Jong et al. [13], only one of 9 (11%) lupin-sensitized patients with a suspected food allergy experienced significant symptoms when challenged with lupin flour. Hence, a clinical lupin allergy in subjects with lupin sensitization seems to be uncommon [6,11,13]. Taking into account that in the study of de Jong et al. [13], the low prevalence of a lupin allergy of 0.3–0.8% was observed in predisposed subjects, the prevalence rate might be even lower in the overall population. To date, the use of lupin as ingredient in German food products is relatively moderate, mainly limited to flour substitutes in bakery, and pasta products, though the availability of lupin-fortified food products is suspected to increase on the market [8]. Thus, with augmented exposition to lupin the prevalence of lupin sensitization and lupin allergy could increase over the next years. However, according to de Jong et al. [13], despite a relatively high consumption of lupin flour, in the Netherlands there is a low prevalence of reported anaphylactic reactions to lupin flour.

Taken altogether, the proportion of clinically relevant lupin allergies does not exceed one-third of the lupin-sensitized subjects, as shown in many though not all studies focusing on lupin allergy [6,11,13,14]. Based on our results, showing a lupin sensitization rate of 4% in the total cohort, we thus suppose that to date the prevalence of a lupin allergy is rather low in the overall German population. Nonetheless, in cases of a lupin allergy, clinical signs seem to be severe, similar to those of a peanut allergy [14]. A lupin allergy can trigger symptoms ranging from mild local reactions to systemic anaphylaxis [15]. As reviewed by Jappe and Vieths [16], the predominant symptoms of a lupin allergy observed in food challenge tests were asthma, urticaria, rhinitis, and abdominal discomfort. The eliciting dose of lupin flour (0.5 mg) that induces subjective symptoms is low [16,17]. Thus, the mandatory labeling of the use of lupin in food products is a crucial means to avoid severe reactions. However, overall education is at least as important to raise the awareness of potential risks that are associated with the intake of lupin-containing products, especially in consumers allergic to other legumes.

### Cross-reactivity among legumes

Structural homologies of the allergens among the *Fabaceae* family might be responsible for the high cross-reactivity rate between legumes. In lupin, β-conglutin, PR-10, and α-conglutin seem to cross-react with Ara h 1, Ara h 8, and Ara h 3/4 of peanut [7,17,18]. Apparently, the probability of a cross-reaction rises with increasing phylogenetic relationship [13]. Sanz et al. [17] report that, in particular, peanut, soybean, and pea are likely to cross-react with lupin.

In the present study, of the 6 lupin-sensitized subjects, a relatively high proportion was also sensitized to pea (50%), to peanut (50%), and especially to soybean (83%). All but one lupin-sensitized subject were sensitized to at least one other legume tested. Obviously, sensitization to soybean is very likely in lupin-sensitized subjects. The sensitization rate to lupin in subjects with positive SPT to pea was quite low (25%). The frequency of a sensitization to lupin in subjects with positive SPT to peanut (60%) or soybean (63%), however, was high. This is also supported by an only moderate level of coincidence found in the distributions of positive and negative SPT reactions between lupin and pea (kappa = 0.476, *P* < 0.0001), and high observed kappa coefficients between lupin and peanut (kappa = 0.853, *P* < 0.0001) and between lupin and soybean (kappa = 0.697, *P* < 0.0001).

The current data in the literature concerning the frequency of cross-reactivities between lupin and other legumes has not yet been fully clarified. Moneret-Vautrin et al. [4] found 11 of 24 (44%) peanut-allergic patients to be sensitized to lupin flour. In the study of Shaw et al. [6], 16 of 47 (34%) peanut-allergic subjects were sensitized to lupin. Peeters et al. [14] studied 37 peanut-sensitized subjects and found a high rate of cross-sensitization between peanut and lupin (26 of 37, 70%). In the study of Gayraud et al. [12], 7 out of 48 (15%) peanut-allergic adults were sensitized to lupin. Fiocchi et al. [19] assessed lupin tolerance in peanut-allergic children and found a lupin sensitization rate of 67% (8 of 12).

According to Moneret-Vautrin et al. [4] and Jappe and Vieths [16], the risk of cross-reactivity between lupin and peanut is high, whereas that between lupin and other legumes, such as soybean, lentils, pea, and beans, is only rarely relevant. In contrast, our results show that the risk of cross-reactivity between lupin and soy might be comparably high or even higher than that between lupin and peanut.

### Limitations of the study

The present study has a few limitations. First, the cohort was too small to draw decisive conclusions about lupin sensitization concerning the overall population. However, the study helps to make preliminary estimations of the relevance of a sensitization to lupin in healthy as well as in predisposed atopic subjects.

Moreover, the predictive value of the SPT for a clinically relevant allergy is limited. Both false-negative and false-positive SPT results may occur [20]. As reported by Verstege et al. [21], SPTs are high in sensitivity and relatively low in specificity. Thus, a sensitization determined by a positive SPT does not prove a clinically relevant allergy. Since, in the present study, no oral food challenges were carried out, the allergy prevalence to lupin in the examined cohort could not be assessed.

Finally, we only used one protein compound from lupin (*Lupinus albus*). Different species or even cultivars as well as different processing and extraction methods may be accompanied by a varying allergic potential of lupin due to differences in epitope structure or availability of the allergen [11]. According to Lindvik et al. [11], the allergen preparation used has considerable influence on the SPT results. This also might explain the discrepancy of observed SPT positivities in the present study and the results of previous studies. For this reason, there is an increasing demand for standardized allergen solutions for lupin to ensure a more precise and comparable estimate of prevalence rates in Germans as well as in other nationalities.

## Conclusions

We have demonstrated that the 2% prevalence of a lupin sensitization is comparable to or even lower than that of other legumes, such as pea (2%), peanut (2%), and soybean (3%) in non-atopic subjects. Since a clinically relevant allergy does presumably not concern more than one-third of the sensitized subjects, to date, lupin allergy is suspected to be relatively uncommon in the overall German population. However, in the present study, the prevalence rate for lupin sensitization in atopic subjects was 6%. Thus, there is a clear risk of a lupin allergy in predisposed subjects. In particular, subjects with existing sensitization or allergy to legumes, others than lupin, are at higher risk for a sensitization or allergy to lupin due to cross-reactivity. Consequently, this study reinforces the need of mandatory labeling of lupin on the ingredients list of food products for consumer protection as advised by the European Commission Directive 2006/142/EC [2].

The present study contributes to the current literature as it provides preliminary indications with respect to the frequency of lupin sensitization in German subjects, thereby comparing the prevalence rate to that of sensitization to other legumes. The potential of lupin to elicit sensitization and/or allergy in German subjects has to be fully assessed in further studies including additional examinations, such as oral food challenges in order to be able to draw comprehensive conclusions about the distribution and severity of a lupin allergy in the population.
